# Legionnaires’ disease in the time of COVID-19

**DOI:** 10.1186/s41479-020-00080-5

**Published:** 2021-01-06

**Authors:** Kelsie Cassell, J Lucian Davis, Ruth Berkelman

**Affiliations:** 1 Department of Epidemiology of Microbial Diseases, Yale School of Public Health, New Haven, CT USA; 2 Pulmonary, Critical Care, and Sleep Medicine Section, Yale School of Medicine, New Haven, CT USA; 3 Rollins School of Public Health, Emory University, Atlanta, GA USA

**Keywords:** Legionnaires’ disease, Epidemiology, COVID-19

## Abstract

Due to similarities in initial disease presentation, clinicians may be inclined to repeatedly test community-acquired pneumonia cases for COVID-19 before recognizing the need to test for Legionnaires’ disease. Legionnaires’ disease is an illness characterized by pneumonia that has a summer/early fall seasonality due to favorable conditions for *Legionella* growth and exposure. *Legionella* proliferate in warm water environments and stagnant sections of indoor plumbing and cooling systems. During the ongoing pandemic crisis, exposures to aerosolized water from recently reopened office or retail buildings should be considered as an epidemiologic risk factor for *Legionella* exposure and an indication to test. The majority of Legionnaires’ disease cases occurring each year are not diagnosed, and some experts recommend that all patients hospitalized with community-acquired pneumonia without a known etiology be tested for *Legionella* infection. Proper diagnosis can increase the likelihood of appropriate and timely antibiotic treatment, identify potential clusters of disease, and facilitate source attribution.

As SARS-CoV-2 continues to sweep through the world’s population, healthcare providers should be on heightened alert for another potential cause of pneumonia with similar symptoms: Legionnaires’ disease. Public health professionals have recognized that due to the similarities in initial disease presentation, clinicians may repeatedly test for coronavirus disease 2019 (COVID-19) before recognizing the need to test for Legionnaires’ disease. Legionnaires’ disease is a common cause of community-acquired pneumonia with ~ 10% mortality; most patients require hospitalization with some progressing to acute respiratory failure leading to intensive care unit admission, similar to COVID-19 [1]. Infections are due to inhalation of aerosols containing *Legionella*. As buildings reopen and previously stagnant plumbing and cooling systems return to use, many additional cases could present to emergency departments in the coming months. Over the past two decades, U.S. incidence of Legionnaires’ disease has increased over five-fold to more than 3.0 cases per 100,000 population in 2018, with most cases occurring in the summer and early autumn (Fig. 1). Yet, Legionnaires’ disease remains vastly underdiagnosed with the true number of cases estimated to be more than 50,000 per year [2].

**Fig. 1:**
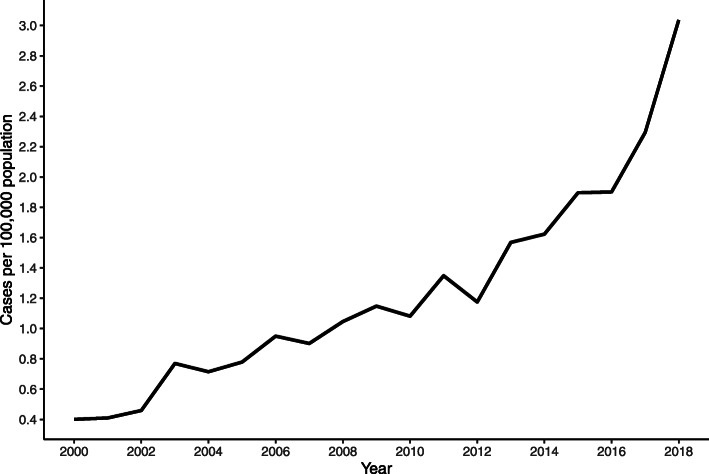
Incidence of Legionnaires’ disease in the United States, 2000 – 2018. Note: Data from the CDC National Notifiable Diseases Surveillance System, 2000 – 2018

Several reports have underscored the need for caution when re-opening buildings [3]. Aerosolization of waterborne *Legionella* can occur from both indoor and outdoor sources, including showers, gardening hoses, fountains, hot tubs, and cooling towers. Biofilms proliferate in water pipes under low-flow conditions, especially where disinfectant levels are inadequate or when building occupancy is low. Garrison et al. [4] noted that building-related Legionnaires’ disease outbreaks investigated between 2000 and 2014 in North America included outbreaks attributed to low occupancy, closures of hospital wards, and water stagnation due to water flow disruptions (e.g. construction, water main break) [4]. As states relax lockdowns and physical distancing measures in response to COVID-19, the reopening of commercial buildings – and more importantly, the taps of their dormant plumbing systems – has the potential to expose large numbers of people to stagnant water containing *Legionella spp.* Additionally, these reopenings coincide with the annual seasonal peak of Legionnaires’ disease cases in the Northern hemisphere [5]. Over the last few months, the CDC, the EPA, and multiple professional societies (e.g. AIHA, ASHRAE) have issued guidance on safely reopening buildings in order to prevent *Legionella* growth and transmission linked to pandemic response measures [3, 6–8].

Pneumonia is the most common manifestation of both Legionnaires’ disease and COVID-19, and initial presentation for both may include fever, headache, confusion, dyspnea, nausea, and diarrhea. Individual risk factors for both Legionnaires’ disease and severe COVID-19 include older age, diabetes, and chronic lung disease. The incubation period for Legionnaires’ disease is about 5 to 6 days but may range from 2 to 14 days, similar to COVID-19. Travel with overnight stays and healthcare exposure (e.g. hospitals, long-term care facilities) are known major risk factors for Legionnaires’ disease [1]. During the ongoing pandemic crisis, exposures to aerosolized water from recently reopened office or retail buildings should also be considered as new risk factors for *Legionella* exposure. Additionally, changing behaviors in the home and for recreational activities during COVID-19 should be considered (e.g. gardening).

Because clinical manifestations may be indistinguishable between COVID-19 and Legionnaires’ disease, targeted microbiologic testing for both *Legionella* and SARS-CoV2 are essential. The American Thoracic Society (ATS) and the Infectious Diseases Society of America (IDSA) guidelines for community-acquired pneumonia recommend *Legionella* urinary antigen testing (UAT) for severe disease in adults or those with epidemiological indications, in addition to sending sputum and other lower respiratory tract specimens for PCR and culture [9]. It is important to note that the UAT has a sensitivity around 70% and a 99% specificity but this is for *Legionellapneumophila* serogroup 1 and cannot reliably detect other pathogenic species and serogroups of *Legionella* that cause disease [10]. Historically, epidemiological indication referred to recent travel (e.g. hotels, cruises) or history of recent hospitalization or residence in a long-term care facility. Currently, it is important to consider other risk factors as well, including returning to work in reopened office buildings or patronizing businesses or other buildings that had been shuttered. The Centers for Disease Control and Prevention in Atlanta closed multiple buildings in August due to presence of Legionella in the buildings water systems, potentially linked to the long term building closure during the pandemic [11]. 

Some experts recommend that all patients hospitalized with pneumonia and without a known etiology be tested with UAT [2], however, IDSA/ITS guidelines recommend only severe CAP be tested with the UAT. This test is widely considered to have relatively low cost, with the ability to reduce discordant antibiotic therapy, and is of greater applicability in higher prevalence areas of the US [12, 13]. Of note, coinfection with *Legionella* and SARS-CoV2 has been documented [14]. Because the UAT cannot detect *Legionella* caused by non-*Legionella pneumophila* serogroup 1, and because morbidity and mortality due to *Legionella* are high, ATS/IDSA guidelines also recommend early empiric antimicrobial therapy with a fluoroquinolone or macrolide such as azithromycin and levofloxacin [1].

Because empiric treatment of Legionnaires’ disease is not always effective, early testing including UAT could improve the clinical and public health response. Like COVID-19, public health officials require a laboratory diagnosis to investigate cases, which could lead to identification of the exposure source. Such actions will reduce morbidity and mortality from this severe and increasingly common disease.

## Data Availability

The datasets used to generate Fig. 1 are available from CDC National Notifiable Diseases Surveillance System, 2000–2018.

## Abbreviations

COVID-19: Coronavirus disease 2019
CDC: Centers for Disease Control and Prevention
EPA: Environmental Protection Agency
AIHA: American Industrial Hygiene Association
ASHRAE: American Society of Heating, Refrigerating, and Air Conditioning Engineers
UAT: Legionella urinary antigen testing
ATS/IDSA: The American Thoracic Society (ATS)/The Infectious Diseases Society of America
